# Association of ZC3HAV1 single nucleotide polymorphisms with the susceptibility of Vogt-Koyanagi-Harada Disease

**DOI:** 10.1186/s12920-023-01546-3

**Published:** 2023-05-23

**Authors:** Qiuying Wu, Zhenyu Zhong, Chunya Zhou, Qingfeng Cao, Guannan Su, Peizeng Yang

**Affiliations:** 1 The First Affiliated Hospital of Chongqing Medical University, Chongqing Key Laboratory of Ophthalmology, Chongqing Eye Institute, Chongqing Branch (Municipality Division) of National Clinical Research Center for Ocular Diseases, Chongqing, China; 2 Department of Ophthalmology & Optometry, The School of Medical Technology and Engineering, Fujian Medical University, Fuzhou, P. R. China

**Keywords:** ZC3HAV1, Vogt-Koyanagi-Harada disease, Single nucleotide polymorphisms, Autoimmune, Genetic susceptibility

## Abstract

**Background:**

Polymorphisms of genes related to the immune response have been reported to confer susceptibility to Vogt-Koyanagi-Harada (VKH) disease. This study was carried out to determine whether zinc finger CCCH-type containing antiviral 1 (ZC3HAV1) and tripartite motif-containing protein 25 (TRIM25) genetic polymorphisms are associated with this disease.

**Methods:**

A total of 766 VKH patients and 909 healthy individuals were enrolled in this two-stage case-control study. Thirty-one tag single nucleotide polymorphisms (SNPs) of ZC3HAV1 and TRIM25 were genotyped by MassARRAY System and iPLEX Gold Genotyping Assay. Allele and genotype frequencies were analyzed by the χ^2^ test or Fisher’s exact test. Cochran-Mantel-Haenszel test was used to assess the pooled odds ratio (OR) in the combined study. A stratified analysis was performed in terms of the major clinical features of VKH disease.

**Results:**

We found a statistically significant increased frequency of the minor A allele of ZC3HAV1 rs7779972 (*P* = 1.50 × 10^− 4^, pooled OR = 1.332, 95%CI = 1.149–1.545) in VKH disease as compared with controls by using the Cochran-Mantel-Haenszel test. The GG genotype of rs7779972 showed a protective association with VKH disease (*P* = 1.88 × 10^− 3^, OR = 0.733, 95%CI = 0.602–0.892). There was no difference regarding the frequency of the remaining SNPs between VKH cases and controls (all *P* > 2.08 × 10^− 3^). The stratified analysis showed no significant association of rs7779972 with the major clinical characteristics of VKH disease.

**Conclusion:**

Our study indicated that the ZC3HAV1 variant rs7779972 might confer susceptibility to VKH disease in Han Chinese.

**Supplementary Information:**

The online version contains supplementary material available at 10.1186/s12920-023-01546-3.

## Background

Vogt-Koyanagi-Harada (VKH) disease is a systemic autoimmune disorder characterized by granulomatous uveitis, with or without extraocular features, including neurological abnormalities, alopecia, tinnitus, and vitiligo [1], [2]. It is one of the most common uveitis entities in China [3]. The specific etiology and pathogenesis of VKH disease remain uncertain, but genetic factors and abnormal immune responses have been shown to be implicated in its development [1], [4], [5]. Polymorphisms of genes related to the immune response have been reported to confer susceptibility to this disease [1]. In addition, several previous studies suggested that viral infection might be an immunological triggering factor for VKH disease [6–9].

Zinc finger CCCH-type containing antiviral 1 (ZC3HAV1), also known as zinc-finger antiviral protein (ZAP) and poly (ADP-ribose) polymerase-13 (PARP13), is an essential intracellular antiviral and pro-inflammatory factor [10, 11]. ZC3HAV1 has been shown to inhibit a wide range of viruses such as human cytomegalovirus (HCMV) [12], influenza A virus (IAV) [13], and severe acute respiratory syndrome coronavirus 2 (SARS-CoV-2) [14]. In addition, studies suggest that ZC3HAV1 is involved in the innate immune response through posttranscriptional RNA regulation [11]. The tripartite motif-containing protein 25 (TRIM25) was found to be required for the antiviral activity of ZC3HAV1 [15–17], implicated in the innate immune response against RNA viruses as well [18]. ZC3HAV1 and TRIM25 were shown to contribute to the up-regulation of type I interferons (IFN) upon viral infection [18–20]. Moreover, polymorphisms in ZC3HAV1 were found to be associated with the susceptibility to multiple sclerosis (MS) in an Italian population [21]. Several studies also indicated that TRIM25 is involved in the development of autoimmune disorders and inflammation [22, 23].

Given the potential link of ZC3HAV1 as well as TRIM25 with autoimmunity, we presume that genetic polymorphisms in ZC3HAV1 and TRIM25 might confer susceptibility to VKH disease. This study was therefore carried out to examine the association between polymorphisms of these two genes and VKH disease.

## Methods

### Participants

The study enrolled a total of 766 patients with VKH disease and 909 healthy individuals from the First Affiliated Hospital of Chongqing Medical University, Chongqing, China between September 2016 and November 2020. This study was carried out by two stages. A cohort containing 384 VKH cases and 384 controls was recruited for the first stage of study. The subjects enlisted in the second stage of study consisted of 382 cases and 525 controls for validation. The diagnosis of VKH disease was made according to both set criteria: the Standardization of Uveitis Nomenclature (SUN) Working Group’s criteria [24] and those proposed by our team [25]. All control subjects were matched for sex, ethnicity, and geographic region with VKH cases. Research design and experimental procedures were conducted according to the Declaration of Helsinki. This study was approved by the Ethical Committee of Chongqing Medical University (No. 2009-201008). Informed consent was obtained from all individual participants included in the study.

### Tag SNPs selection

The selection of the candidate SNPs was carried out according to the data of the Han Chinese in Beijing (CHB) from 1000 Genomes Project (http://grch37.ensembl.org/). We used Haploview 4.2 program to select tag SNPs with a minor allele frequency (MAF) over 0.05 and r^2^ threshold higher than 0.8 (r^2^ > 0.8). Finally, thirty-one tag SNPs (22 variants of ZC3HAV1 and 9 variants of TRIM25) were included for this study (Supplementary Table S1).

### DNA extraction and genotyping

Genomic DNA was extracted from the collected peripheral blood samples of all participants by using the QIAamp DNA Blood Mini Kit (Qiagen, Valencia, CA, USA). The concentration and purity of all DNA samples were examined on the Nanodrop 2000 (Thermo Fisher Scientific, Wilmington, Delaware, USA). The primers of all selected SNPs were designed using the MassArray Assay Design software (Sequenom) and were shown in Supplementary Table S2. All DNA samples and primers were stored at − 20℃. Polymerase chain reaction (PCR) was performed with a GeneAmp PCR System 9700 instrument (ABI, Foster City, California, USA). Genotypes of the 31 tag SNPs were identified through MassARRAY System (Sequenom, San Diego, California, USA) and iPLEX Gold Genotyping Assay, then analyzed the experimental data via TYPER V.4.0 software. In the second stage, one statistically significant SNP in the first stage was further genotyped using this method. All experimental steps were strictly conducted based on the manufacturer’s protocol.

### Statistical analysis

The chi-square (χ^2^) test was chosen to analyze the Hardy-Weinberg equilibrium (HWE) in healthy controls. The demographic data of subjects were compared by Mann-Whitney U test or χ^2^ test. The Quanto software was used to calculate the statistical power with the following parameters: a minor allele frequency of 0.058, the dominant model, a prevalence of VKH disease in Asian of 0.001% [26] and an odds ratio (OR) of 2.0. Allele and genotype frequencies were compared between VKH case and control groups by the χ^2^ test or Fisher’s exact test, as appropriate. Cochran-Mantel-Haenszel test was applied to estimate the pooled OR accounting for the stratification factor (two stages of study) [27]. All the statistical analyses were performed with the IBM SPSS Statistics V.25.0 software. The Bonferroni correction method was applied to adjust the p values for multiple comparisons. *P* < 2.08 × 10^− 3^ (0.05/24) was considered to have statistical significance, since twenty-four SNPs were included to be tested in this study. Three genetic inheritance models were additionally constructed to assess the genotype frequencies, including dominant, recessive, and co-dominant [28]. Logistic regression analyses were executed to adjust the odds ratios for sex and age.

## Results

### Clinical features

Demographic data and clinical features of enrolled VKH cases and healthy controls are presented in Table 1. The VKH case group consisted of 400 men (52.2%) and 366 women (47.8%) in total, with a median age (interquartile range [IQR]) of 42 (31 to 50) years. The median age (IQR) of the control group was 39 (32 to 46) years, including 450 men (49.5%) and 459 women (50.5%). All participants were Han Chinese.

**Table 1:** Clinical features of VKH patients and healthy controls enrolled in the study

Clinical features	First Stage		Second stage		Total	
	VKH (n = 384)	%	VKH (n = 382)	%	VKH (n = 766)	%
Age, median (IQR)	44 (32–53)		39.5 (29–47)		42 (31–50)	
Male	199	51.8	201	52.6	400	52.2
Female	185	48.2	181	47.4	366	47.8
Uveitis	384	100.0	382	100.0	764	100.0
Sunset glow fundus	181	47.1	190	50.0	371	48.6
Headache	193	50.3	175	46.1	368	48.2
Tinnitus	171	44.5	166	43.5	337	44.0
Vitiligo	39	10.2	51	13.4	90	11.8
Alopecia	106	27.6	128	33.7	234	30.6
Poliosis	102	26.6	114	30.0	216	28.3
Dysacusia	148	38.5	118	30.9	266	34.7
Healthy controls	384		525		909	
Age, median (IQR)	38 (31–46)		40 (33–46)		39 (32–46)	
Male	183	47.7	267	50.9	450	49.5
Female	201	52.3	258	49.1	459	50.5

VKH: Vogt-Koyanagi-Harada Disease

IQR: Interquartile range

### Genotyping results of tested SNPs in the first stage

Among 31 SNPs genotyped in the first stage, seven SNPs were not included in the analysis because rs77429073, rs17133451, rs10272189, rs205494 could not be genotyped successfully with the assay, rs528478132 and rs200986947 were not polymorphic in this cohort, and rs55946764 was deviated from the Hardy–Weinberg equilibrium (*P* < 0.01). The statistical power was 93.01% according to the sample size in the first stage. A significant higher frequency of the ZC3HAV1 rs7779972 minor A allele (*P* = 0.001, OR = 1.429, 95%CI = 1.148–1.778) was found in VKH patients as compared with the controls (Table 2). No statistically significant association was observed between the other 23 SNPs with the risk of VKH (all *P* > 2.08 × 10^− 3^) (Supplementary Table S3).

**Table 2:** Allelic distributions of the rs7779972 in patients with VKH and healthy controls

Allele	VKH	Controls	P*	OR (95% CI) *
	n (%)	n (%)		
First				
A	262(35.2)	210(27.6)	**0.001**	1.429(1.148–1.778)
G	482(64.8)	552(72.4)	**0.001**	0.700(0.562–0.871)
Second				
A	258(35.3)	312(30.4)	**0.028**	1.254(1.025–1.535)
G	472(64.7)	716(69.6)	**0.028**	0.797(0.652–0.975)
Combined			*P*†	pooled OR (95% CI) †
A	520(35.3)	522(29.2)	**1.50 × 10** ^**− 4**^	1.332(1.149–1.545)
G	954(64.7)	1268(70.8)	**1.50 × 10** ^**− 4**^	0.751(0.647–0.871)

VKH: Vogt-Koyanagi-Harada Disease; OR: odds ratio; 95% CI: 95% confidence interval.

* χ^2^ test.

† Cochran-Mantel-Haenszel test.

*P* < 2.08 × 10^− 3^ (0.05/24) indicates statistical significance for each variant tested. Values with statistical significance are in boldface.

### Genotyping results of tested SNPs in the second stage and combined study

In the second stage of the study for further replication and validation of first-stage findings, rs7779972 was genotyped in another independent cohort. A higher frequency of the minor A allele for rs7779972 was consistently observed (*P* = 0.028, OR = 1.254, 95%CI = 1.025–1.535) in VKH cases as compared with the controls.

There was similar homogeneity of OR in these two stages cohort (*P* = 0. 391), we therefore combined the data of the two stages, the rs7779972 minor A allele was significantly associated with an increased risk of VKH disease through the Cochran-Mantel-Haenszel test (*P* = 1.50 × 10^− 4^, pooled OR = 1.332, 95%CI = 1.149–1.545, Table 2). The significant association between the minor A allele of rs7779972 and VKH disease was further confirmed by logistic regression analysis (*P*_adj_ = 2.29 × 10^− 4^, OR = 1.321, 95%CI = 1.139–1.532) after adjusting the gender and age in the combined populations.

In addition, we found a protective association between the GG genotype and VKH disease (*P* = 1.88 × 10^− 3^, OR = 0.733, 95%CI = 0.602–0.892) in the combined subjects (Table 3). Three genetic models were subsequently applied to explore the genotypic association between rs7779972 and VKH risk by logistic regression analysis. Subjects carrying the minor A allele were more susceptible to VKH disease as compared to those with the GG homozygous genotype under the co-dominant and dominant models (Table 4). Similarly, the AG and GG genotypes instead of the AA genotype showed a protective association with VKH disease under a recessive model (*P* = 0.003, OR = 0.609, 95%CI = 0.438–0.846).

**Table 3:** Genotype distributions of rs7779972 between VKH case and control groups

Genotype	VKH	Controls	*P*	OR (95% CI)
	n (%)	n (%)		
First				
AA	47(12.6)	25(6.6)	0.005	2.059(1.239–3.422)
AG	168(45.2)	160(42.0)	0.381	1.138(0.853–1.518)
GG	157(42.2)	196(51.4)	0.011	0.689(0.517–0.919)
Second				
AA	44(12.1)	45(8.7)	0.110	1.429(0.921–2.216)
AG	170(46.6)	222(41.2)	0.320	1.147(0.876–1.502)
GG	151(41.4)	247(48.1)	0.050	0.763(0.582-1.000)
Combined				
AA	91(12.3)	70(7.8)	2.28 × 10^− 3^	1.660(1.196–2.305)
AG	338(45.9)	382(42.7)	0.198	1.138(0.935–1.384)
GG	308(41.8)	443(49.5)	**1.88 × 10** ^**− 3**^	0.733(0.602–0.892)

VKH: Vogt-Koyanagi-Harada Disease; OR: Odd ratios; 95% CI :95% confidence interval

*P:* P-value which was calculated by χ^2^ test lower than 2.08 × 10^− 3^ (0.05/24) has statistical significance, and indicated by boldface

**Table 4:** Genotype distributions of rs7779972 between VKH case and control groups under different inheritance models

Genotype	First stage		Second stage		Combined	
	*P*	OR (95% CI)	*P*	OR (95% CI)	*P*	OR (95% CI)
Co-dominant						
GG	-	1	-	1	-	1
AG	0.113	1.280(0.943–1.739)	0.121	1.254(0.942–1.668)	**0.021**	1.275(1.037–1.568)
AA	**0.002**	2.295(1.343–3.922)	**0.038**	1.633(1.027–2.596)	**4.64 × 10** ^**− 4**^	1.852(1.311–2.614)
Dominant						
GG	-	1	-	1	-	1
AA + AG	**0.019**	1.417(1.059–1.897)	**0.047**	1.317(1.004–1.728)	**0.002**	1.365(1.121–1.662)
Recessive						
AA	-	1	-	1	-	1
AG + GG	**0.007**	0.491(0.293–0.822)	0.093	0.686(0.441–1.065)	**0.003**	0.609(0.438–0.846)

VKH: Vogt-Koyanagi-Harada Disease; OR: Odd ratios; 95% CI :95% confidence interval

*P:* Adjusted by age and gender. P-value lower than 0.05 has statistical significance, and indicated by boldface.

### Stratified analysis of the tested SNP with major clinical characteristics of patients with VKH disease

Stratified analysis was executed to estimate whether rs7779972 has an association with the major clinical characteristics of VKH disease, including sunset glow fundus, headache, tinnitus, dysacusia, alopecia, poliosis, and vitiligo. Our results showed that there was no association between the primary clinical manifestations of VKH disease and rs7779972 (Supplementary Table S4).

### Allele frequency analysis of the tested SNP in different populations

We additionally searched for the genetic data of rs7779972 in different populations from the ENSEMBL database. We treated those populations as controls and compared them with VKH cases, respectively. The results showed no significant association between rs7779972 and VKH disease in different East Asian populations (Supplementary Table S5).

## Discussion

In this case-control association study, we examined the association of ZC3HAV1 and TRIM25 genetic polymorphisms with VKH disease in Han Chinese, and observed that the minor A allele and GG genotype of ZC3HAV1 rs7779972 was significantly associated with VKH disease. While rs7779972 did not show any significant association with the primary clinical features of VKH disease.

There was no significant association between rs7779972 and VKH disease in terms of the different populations’ genetic data from the ENSEMBL database. But it is worth noting that the distribution of rs7779972 deviated from the HWE in East Asian population (*P* < 0.05). In addition, the number of subjects in each population was less than that in our study (n = 909). Geographical factor and statistical power should also be taken into consideration. Therefore, the results need to be further confirmed in various populations with sufficient sample size in the future.

The clinical manifestations presented in the prodromal stage of VKH disease are similar to those caused by viral infection symptoms like fever and headache [1]. It has been reported that infective triggers such as CMV [29], Epstein–Barr virus (EBV) [30], IAV [9] and SARS-CoV-2 [6, 8] may be responsible for the onset or development of VKH disease by molecular mimicry and cross reactivity. However, there is a lack of exact experimental proof to support this hypothesis. It has been shown that most viruses as mentioned above are potently inhibited by ZC3HAV1. ZC3HAV1 was found to diminish levels of HCMV mRNA and protein, and inhibit the progression of viral infection by mediating degradation of specific viral transcripts [31], [32]. ZC3HAV1 was also regarded as an interferon-stimulated gene (ISG). A previous study found that it inhibited IAV replication through regulating type I interferon response [33]. In addition, ZC3HAV1 was reported to promote the degradation of cytosine–phosphate–guanine (CpG) containing viral RNAs to inhibit SARS-CoV-2 and conduce to the antiviral effect of IFNs [34]. Although there is no direct evidence to show an inhibition effect of ZC3HAV1 on EBV, the protective allele of MS risk SNP rs10271373 enhanced the expression of ZC3HAV1 in EBV infected B cells [35]. On the other hand, several studies have revealed that ZC3HAV1 affects the expression of pro-inflammatory cytokines involved in the nuclear factor kappa-B (NF-κB) pathway, such as IFN-β, interleukin-6 (IL-6), and tumor necrosis factor (TNF) [19], [33]. These cytokines were directly or indirectly involved in the development of VKH disease [36–41]. All of those results stated above stimulated us to investigated whether the polymorphisms were associated with the development of VKH disease. Further experimental studies are required to confirm the possible mechanisms.

As far as we know, there are few studies concerning the genetic polymorphism of ZC3HAV1. We analyzed seventeen tag SNPs in this study which represent most SNPs of this gene. The variant associated with VKH disease identified in our study was rs7779972, located in the intron of ZC3HAV1. The minor A allele of rs7779972 was a risk allele which confers genetic susceptibility to VKH disease. While the GG genotype showed a protective association with VKH disease. The biological function of this locus is presently uncertain. No association is found between rs7779972 with other diseases. An Italian cohort study indicated an association of the ZC3HAV1 rs3735007 polymorphism with MS [21], an autoimmune disorder which may have a common immune pathogenesis with VKH disease [42], [43]. However, this association was not replicated in a population from Belgium [21]. No significant difference in the genotype distributions of rs3735007 was found in our study (Supplementary Table S3). This discrepancy may be due to the distinct geographic locations, specific disease mechanisms, and ethnic populations.

A previous study has demonstrated upregulation of TRIM25 in muscle samples from dermatomyositis patients [22]. TRIM25 is also implicated in the pathogenesis of neuroinflammation [23]. Polymorphisms in TRIM25 have been reported to be associated with immune responses to measles vaccine [44]. In this study, we did not show any association of TRIM25 gene polymorphism with VKH disease in Han Chinese. We cannot rule out the possibility that other untested TRIM25 variants affect VKH disease susceptibility.

There are some limitations in our study. We only recruited individuals from a Chinese Han population, the finding therefore may need to be confirmed in other ethnic populations. Second, we examined only one widespread uveitis entity, it remains to be seen whether the genetic polymorphisms of ZC3HAV1 may confer susceptibility to other types of uveitis. Last, the possible biological function of rs7779972 is unclear, further functional experiments are necessary to investigate how this variant plays a role in the pathogenesis of VKH disease.

## Conclusions

In summary, this study performed in Han Chinese reveals that rs7779972 of ZC3HAV1 has a significant association with VKH disease, thus providing a novel insight into the etiology and pathogenesis of this disease.

## Electronic supplementary material

Below is the link to the electronic supplementary material.

Supplementary Material 1

## Data Availability

Data will be available from the corresponding author on reasonable request.

## Abbreviations

ZC3HAV1: Zinc finger CCCH-type containing antiviral 1.
TRIM25: tripartite motif-containing protein 25
VKH disease: Vogt-Koyanagi-Harada disease
SNP: single nucleotide polymorphism
HCMV: human cytomegalovirus
IAV: influenza A virus
SARS-CoV-2: severe acute respiratory syndrome coronavirus 2
IFN: interferon
MS: multiple sclerosis
SUN: Standardization of Uveitis Nomenclature
CHB: Han Chinese in Beijing
MAF: minor allele frequency
HWE: Hardy-Weinberg equilibrium
OR: odds ratio
CI: confidence interval
IQR: interquartile range
EBV: Epstein–Barr virus
CpG: cytosine–phosphate–guanine
NF-κB: nuclear factor kappa-B
IL-6: interleukin-6
TNF: tumor necrosis factor
