# Supplementary material for: Association of ZC3HAV1 single nucleotide polymorphisms with the susceptibility of Vogt-Koyanagi-Harada Disease
Source: BMC Med Genomics. 2023 May 23;16:113. doi: 10.1186/s12920-023-01546-3 (PMC10204004; doi:10.1186/s12920-023-01546-3)
Supplement: Supplementary file 1 — Supplementary Material 1 [file 12920_2023_1546_MOESM1_ESM.docx]

**Supplementary Table S1. Detailed information of the 31 tag SNPs**

| Gene | SNP | Location | Allele | MAF in CHB  Freq.(count) | HWE  in this study |
| --- | --- | --- | --- | --- | --- |
| ZC3HAV1 | rs3735007 | chr7:138732497 | G/A | A: 0.369 (76) | 0.17 |
|  | rs3800603 | chr7:138755004 | A/G | G: 0.330 (68) | 0.74 |
|  | rs4728460 | chr7:138779517 | A/G | G: 0.374 (77) | 0.47 |
|  | rs6467822 | chr7:138773011 | C/T | T: 0.320 (66) | 0.53 |
|  | rs7779972 | chr7:138746752 | A/G | A: 0.325 (67) | 0.32 |
|  | rs7782919 | chr7:138735270 | C/T | T: 0.068 (14) | 0.22 |
|  | rs7790567 | chr7:138776500 | C/G | G: 0.461 (95) | 0.26 |
|  | rs7812093 | chr7:138740480 | C/T | C: 0.204 (42) | 0.73 |
|  | rs10262217 | chr7:138761308 | T/A | A: 0.476 (98) | 0.84 |
|  | rs12672278 | chr7:138777415 | T/C | C: 0.325 (67) | 0.20 |
|  | rs17133444 | chr7:138742775 | C/T | T: 0.150 (31) | 0.99 |
|  | rs62485905 | chr7:138741586 | T/C | C: 0.243 (50) | 0.66 |
|  | rs73170907 | chr7:138765443 | T/C | C: 0.117 (24) | 0.06 |
|  | rs74348184 | chr7:138744679 | T/C | C: 0.204 (42) | 0.80 |
|  | rs79284778 | chr7:138769933 | A/G | G: 0.126 (26) | 0.20 |
|  | rs79517923 | chr7:138748432 | C/T | T: 0.131 (27) | 0.76 |
|  | rs116871425 | chr7:138773570 | C/T | T: 0.092 (19) | 0.90 |
|  | rs77429073* | chr7:138773764 | C/T | T: 0.126 (26) | - |
|  | rs17133451* | chr7:138742981 | A/C | C: 0.296 (61) | - |
|  | rs55946764 | chr7:138771146 | C/T | T: 0.078 (16) | ＜0.01 |
|  | rs10272189* | chr7:138744100 | C/T | T: 0.301 (62) | - |
|  | rs528478132※ | chr7:138740111 | T/C | C: 0.170 (35) | - |
| TRIM25 | rs2525994 | chr17:54976922 | A/C | C: 0.117 (24) | 0.40 |
|  | rs205500 | chr17:54967404 | T/C | C: 0.112 (23) | 0.31 |
|  | rs7225205 | chr17:54980981 | C/T | T: 0.218 (45) | 0.16 |
|  | rs77107579 | chr17:54979526 | T/C | C: 0.058 (12) | 0.54 |
|  | rs58489644 | chr17:54973541 | A/G | G: 0.175 (36) | 0.84 |
|  | rs138059750 | chr17:54987006 | A/C | C: 0.078 (16) | 0.94 |
|  | rs2525997 | chr17:54971241 | C/A | A: 0.350 (72) | 0.41 |
|  | rs200986947※ | chr17:54977588 | T/A | A: 0.068 (14) | - |
|  | rs205494* | chr17:54984009 | T/C | C: 0.335 (69) | - |

SNP: single nucleotide polymorphism; MAF: minor allele frequency; CHB: Han Chinese in Beijing, China; HWE: Hardy–Weinberg equilibrium.

*: Four SNPs (rs77429073, rs17133451, rs10272189, rs205494) could not be genotyped successfully with the assay.

^※^: Two SNPs (rs528478132, rs200986947) were not polymorphic in this study.**Supplementary Table S2. Primer sequences used in the analysis of restriction fragment length polymorphism (RFLP) in SNPs.**

| SNP | 1st-PCRP | 2nd-PCRP | UEP_SEQ |
| --- | --- | --- | --- |
| ZC3HAV1 |  |  |  |
| rs17133444 | ACGTTGGATGAGGATAGAATAAGGGCTGGG | ACGTTGGATGAGATGCCCATGAACAGTGAC | AACAGTGACGTGTGC |
| rs3735007 | ACGTTGGATGGAGGGCTCGTGTACGTTATA | ACGTTGGATGCGTCGTTATGTTTGTAGCCC | GTTCTGGTTGGAAAGTTTA |
| rs116871425 | ACGTTGGATGCAGCCATAAACGAATAGGTG | ACGTTGGATGACCTTGGCCTCCCAAAGTTC | TGAGCCACCATACCC |
| rs6467822 | ACGTTGGATGGTGTCTACCTAAAACTTGGG | ACGTTGGATGTTCCTGAGTGGATGAAACTG | GGATGAAACTGTTAATTATCCA |
| rs3800603 | ACGTTGGATGTTCCCATGAAAACTGTGGAG | ACGTTGGATGACATCCAACAAACCTGTGTC | tTGTTTGCAGATTGAATGTTAC |
| rs7812093 | ACGTTGGATGTCAGGGTAACCATACCTGAG | ACGTTGGATGGGTAGCTTTAGCTCTATGGG | gTTAGCTCTATGGGATAGAAAAA |
| rs73170907 | ACGTTGGATGAGCCCCACCTAAAAGTTTTG | ACGTTGGATGAGGATCAGCTCTAGGATGAC | AGTCCCCGGCTCTCT |
| rs528478132 | ACGTTGGATGCACTTAACTCTCTGCTCCAC | ACGTTGGATGTTTCTCTAGGCATCCTTGGC | TGGCCTAGTGGCTGG |
| rs74348184 | ACGTTGGATGACCCATTAGCTGTGTGGATT | ACGTTGGATGATGTGTTACCCAAGCAACCG | AGGTATTATTATTAAGCCCATTT |
| rs10262217 | ACGTTGGATGTTTCTGATGAGTCTCCAAGG | ACGTTGGATGCAGGTTCCATCTATACCAGC | CACTGCTGTGTGTCCATTA |
| rs55946764 | ACGTTGGATGGGCTCCCTTTCCATTGTTAG | ACGTTGGATGAAGCTTCAGGCAAGAACCTC | ctgaAAGAACCTCCCCACCC |
| rs7782919 | ACGTTGGATGGATCAATCAACTCTCATGCC | ACGTTGGATGAAACCCCCCCATCTCTAATA | cacTACAAAGTTAGCTGGGC |
| rs79284778 | ACGTTGGATGCAGCTCAAACTTTAGCGGAC | ACGTTGGATGAGTCTGAACCATCAGAGGTG | cttgtCCCAACAGTCTGTGTTTT |
| rs7790567 | ACGTTGGATGCACCTAGGCCTAGGTGCTG | ACGTTGGATGTGGGAAGTTTCAGTGAGGTC | CACGGTGTCTCAAGT |
| rs10272189 | ACGTTGGATGTTTAAACTACAGGAAGGGGC | ACGTTGGATGCTCCCAAAGTGCTGGGATG | ggGCTGGGATGACAGGC |
| rs12672278 | ACGTTGGATGAAGCCAGATAGCCTAATGGG | ACGTTGGATGTCAGACATGAGCACCTTTGG | TGGAAAAGCCTGGAC |
| rs4728460 | ACGTTGGATGAAAATAACCTAACCCTGGGC | ACGTTGGATGTCAGACTGCACGTGGGAATC | AGTAAGATGAAGCCAGTC |
| rs79517923 | ACGTTGGATGGAACCTATACTCCCAGTTAG | ACGTTGGATGCACGCCATCACTCAGGCTA | gagaACTCAGGCTAGAGTGC |
| rs7779972 | ACGTTGGATGATCCCCATCACAGGCTATTG | ACGTTGGATGTGCAATGTGCCTACTGCAAG | gGCAAGCGTTTATTAATATCAA |
| rs62485905 | ACGTTGGATGGTTCACGTTTTATGTTTTCCC | ACGTTGGATGCAGACCCTGCTTAGATACTG | TGCTTAGATACTGATTTGCAATAC |
| rs17133451 | ACGTTGGATGCCAGGCTGTCTTGGAATAGT | ACGTTGGATGCTCCCCCACTAACATGTTTT | TCACTATGTGCCATGC |
| rs77429073 | ACGTTGGATGGCTGTTGTAACAGAGACCCCC | ACGTTGGATGGTCTAGCTGCCCAACAACTCA | aTGAGGCAGGAGAATTG |
| TRIM25 |  |  |  |
| rs138059750 | ACGTTGGATGGAGGTTGAGCCTGCAGTGA | ACGTTGGATGCCCTGTGAGCTACTTCATCT | aCAGGCTGGAGTGCAG |
| rs2525994 | ACGTTGGATGAGATCAAGGTCATGTAGCGG | ACGTTGGATGAGCTATAGGTGGCTGGTATG | AGAGAAAGAATAGTGGGAAA |
| rs7225205 | ACGTTGGATGCTTTGCCACATGACATAACC | ACGTTGGATGTACCCTATAGTATGCCCTCC | CTTCAGTATCGGCAGGA |
| rs200986947 | ACGTTGGATGGAGCAGAGATTGGGCCATTG | ACGTTGGATGAGATACACACCATCATGCCC | cccgGCCCGGCTAATTAAAAAAAA |
| rs58489644 | ACGTTGGATGGCATACATGTGTTACCATTG | ACGTTGGATGGGGACTGACTAATAAGGAGC | GCATGAAGGAATTTTCTGG |
| rs2525997 | ACGTTGGATGAGCCTGAAGTCTAATCCTCG | ACGTTGGATGTCCCTGGATTTTGTGACACG | gggagTTTGTGACACGGAGTAC |
| rs205500 | ACGTTGGATGTGTAATGCATCTCAGGCCTC | ACGTTGGATGTCTGGCACAAGATGCACAAC | AGTGTTCTGAGCCTGCC |
| rs77107579 | ACGTTGGATGCTAGTCTGGATGGCGTTAAG | ACGTTGGATGATGTGCACTCAGATCCTTCG | gCCAACAGACAGGAATGAACAC |
| rs205494 | ACGTTGGATGTGGTTGAAGCTGGGTTACCA | ACGTTGGATGAAGGACAGTAGACTGGAAAAGAGG | tggAAATTAGCCGGGCGT |

**Supplementary Table S3. Genotype and allele frequencies of the examined SNPs except rs7779972 in VKH patients and controls in the first stage.**

| SNPs | Genotype | VKH  n(freq.) | Control  n(freq.) | *P* | OR (95%CI) |
| --- | --- | --- | --- | --- | --- |
| rs17133444 | TT | 12(0.032) | 8(0.021) | 0.377 | 1.500(0.606-3.712) |
|  | TC | 87(0.229) | 94(0.250) | 0.498 | 0.891(0.638-1.244) |
|  | CC | 281(0.739) | 274(0.729) | 0.738 | 1.057(0.765-1.459) |
|  | T | 111(0.146) | 110(0.146) | 0.990 | 0.998(0.750-1.328) |
|  | C | 649(0.854) | 642(0.854) | 0.990 | 1.002(0.753-1.333) |
| rs3735007 | AA | 51(0.134) | 48(0.127) | 0.757 | 1.069(0.700-1.631) |
|  | AG | 199(0.524) | 191(0.504) | 0.587 | 1.082(0.814-1.439) |
|  | GG | 130(0.342) | 140(0.369) | 0.432 | 0.888(0.659-1.195) |
|  | A | 301(0.396) | 287(0.379) | 0.486 | 1.076(0.875-1.323) |
|  | G | 459(0.604) | 471(0.621) | 0.486 | 0.929(0.756-1.142) |
| rs74348184 | TT | 247(0.650) | 224(0.599) | 0.148 | 1.244(0.926-1.671) |
|  | TC | 117(0.308) | 132(0.353) | 0.189 | 0.816(0.602-1.105) |
|  | CC | 16(0.042) | 18(0.048) | 0.690 | 0.869(0.436-1.732) |
|  | T | 611(0.804) | 580(0.775) | 0.174 | 1.188(0.927-1.522) |
|  | C | 149(0.196) | 168(0.225) | 0.174 | 0.842(0.657-1.079) |
| rs7812093 | TT | 226(0.596) | 224(0.604) | 0.835 | 0.969(0.724-1.298) |
|  | TC | 139(0.367) | 130(0.350) | 0.641 | 1.074(0.797-1.447) |
|  | CC | 14(0.037) | 17(0.046) | 0.541 | 0.799(0.388-1.645) |
|  | T | 591(0.780) | 578(0.779) | 0.974 | 1.004(0.787-1.282) |
|  | C | 167(0.220) | 164(0.221) | 0.974 | 0.996(0.780-1.271) |
| rs116871425 | TT | 3(0.008) | 2(0.005) | 1.000 | 1.476(0.245-8.885) |
|  | TC | 68(0.179) | 53(0.142) | 0.169 | 1.316(0.890-1.947) |
|  | CC | 309(0.813) | 318(0.853) | 0.148 | 0.753(0.512-1.107) |
|  | T | 74(0.097) | 57(0.076) | 0.149 | 1.304(0.909-1.871) |
|  | C | 686(0.903) | 689(0.924) | 0.149 | 0.767(0.534-1.101) |
| rs3800603 | AA | 203(0.533) | 177(0.467) | 0.070 | 1.302(0.979-1.731) |
|  | AG | 148(0.388) | 166(0.438) | 0.165 | 0.815(0.610-1.088) |
|  | GG | 30(0.079) | 36(0.095) | 0.426 | 0.814(0.491-1.352) |
|  | A | 554(0.727) | 520(0.686) | 0.079 | 1.219(0.977-1.521) |
|  | G | 208(0.273) | 238(0.314) | 0.079 | 0.820(0.658-1.023) |
| rs6467822 | TT | 56(0.147) | 33(0.087) | 0.011 | 1.801(1.142-2.842) |
|  | TC | 163(0.428) | 166(0.439) | 0.753 | 0.955(0.717-1.273) |
|  | CC | 162(0.425) | 179(0.474) | 0.181 | 0.822(0.618-1.095) |
|  | T | 275(0.361) | 232(0.307) | 0.026 | 1.275(1.030-1.58) |
|  | C | 487(0.639) | 524(0.693) | 0.026 | 0.784(0.633-0.971) |
| rs12672278 | TT | 161(0.424) | 172(0.469) | 0.216 | 0.833(0.624-1.113) |
|  | TC | 166(0.437) | 166(0.452) | 0.670 | 0.939(0.704-1.254) |
|  | CC | 53(0.139) | 29(0.079) | 0.008 | 1.889(1.172-3.045) |
|  | T | 488(0.642) | 510(0.695) | 0.031 | 0.788(0.635-0.978) |
|  | C | 272(0.358) | 224(0.305) | 0.031 | 1.269(1.022-1.575) |
| rs10262217 | AA | 72(0.194) | 93(0.247) | 0.079 | 0.733(0.517-1.038) |
|  | AG | 197(0.531) | 186(0.495) | 0.321 | 1.157(0.868-1.541) |
|  | GG | 102(0.275) | 97(0.258) | 0.600 | 1.091(0.788-1.509) |
|  | A | 341(0.460) | 372(0.495) | 0.174 | 0.869(0.709-1.064) |
|  | G | 401(0.540) | 380(0.505) | 0.174 | 1.151(0.940-1.411) |
| rs79284778 | AA | 326(0.867) | 314(0.835) | 0.219 | 1.287(0.860-1.927) |
|  | AG | 46(0.122) | 57(0.152) | 0.243 | 0.780(0.514-1.185) |
|  | GG | 4(0.011) | 5(0.013) | 1.000 | 0.798(0.213-2.995) |
|  | A | 698(0.928) | 685(0.911) | 0.218 | 1.264(0.870-1.837) |
|  | G | 54(0.072) | 67(0.089) | 0.218 | 0.791(0.544-1.149) |
| rs7782919 | TT | 1(0.003) | 4(0.011) | 0.208 | 0.237(0.026-2.132) |
|  | TC | 55(0.148) | 49(0.138) | 0.694 | 1.087(0.717-1.647) |
|  | CC | 316(0.849) | 303(0.851) | 0.950 | 0.987(0.657-1.483) |
|  | T | 57(0.077) | 57(0.08) | 0.807 | 0.953(0.650-1.398) |
|  | C | 687(0.923) | 655(0.92) | 0.807 | 1.049(0.716-1.537) |
| rs4728460 | AA | 136(0.364) | 150(0.397) | 0.349 | 0.869(0.647-1.166) |
|  | AG | 163(0.436) | 171(0.452) | 0.648 | 0.935(0.701-1.247) |
|  | GG | 75(0.201) | 57(0.151) | 0.073 | 1.413(0.967-2.063) |
|  | A | 435(0.582) | 471(0.623) | 0.100 | 0.841(0.684-1.034) |
|  | G | 313(0.418) | 285(0.377) | 0.100 | 1.189(0.967-1.462) |
| rs79517923 | TT | 9(0.025) | 8(0.021) | 0.750 | 1.169(0.446-3.065) |
|  | TC | 67(0.184) | 99(0.263) | 0.010 | 0.633(0.446-0.899) |
|  | CC | 288(0.791) | 270(0.716) | 0.018 | 1.502(1.071-2.105) |
|  | T | 85(0.117) | 115(0.153) | 0.044 | 0.735(0.544-0.992) |
|  | C | 643(0.883) | 639(0.847) | 0.044 | 1.361(1.008-1.839) |
| rs62485905 | TT | 198(0.532) | 206(0.541) | 0.817 | 0.967(0.726-1.287) |
|  | TC | 155(0.417) | 146(0.383) | 0.349 | 1.150(0.859-1.539) |
|  | CC | 19(0.051) | 29(0.076) | 0.160 | 0.653(0.360-1.187) |
|  | T | 551(0.741) | 558(0.732) | 0.714 | 1.044(0.830-1.313) |
|  | C | 193(0.259) | 204(0.268) | 0.714 | 0.958(0.762-1.205) |
| rs7790567 | GG | 92(0.251) | 90(0.245) | 0.848 | 1.033(0.739-1.445) |
|  | GC | 177(0.482) | 173(0.47) | 0.741 | 1.050(0.786-1.403) |
|  | CC | 98(0.267) | 105(0.285) | 0.579 | 0.913(0.660-1.261) |
|  | G | 361(0.492) | 353(0.48) | 0.640 | 1.050(0.856-1.288) |
|  | C | 373(0.508) | 383(0.52) | 0.640 | 0.952(0.776-1.168) |
| rs73170907 | TT | 289(0.798) | 314(0.837) | 0.170 | 0.769(0.528-1.120) |
|  | TC | 67(0.185) | 55(0.147) | 0.161 | 1.321(0.895-1.952) |
|  | CC | 6(0.017) | 6(0.016) | 0.951 | 1.037(0.331-3.244) |
|  | T | 645(0.891) | 683(0.911) | 0.204 | 0.801(0.568-1.128) |
|  | C | 79(0.109) | 67(0.089) | 0.204 | 1.249(0.886-1.759) |
| rs7225205 | TT | 21(0.056) | 13(0.035) | 0.158 | 1.656(0.817-3.359) |
|  | TC | 110(0.293) | 137(0.364) | 0.038 | 0.724(0.533-0.983) |
|  | CC | 244(0.651) | 226(0.601) | 0.160 | 1.236(0.919-1.662) |
|  | T | 152(0.203) | 163(0.217) | 0.502 | 0.918(0.716-1.178) |
|  | C | 598(0.797) | 589(0.783) | 0.502 | 1.089(0.849-1.396) |
| rs2525994 | AA | 278(0.732) | 264(0.706) | 0.433 | 1.136(0.827-1.560) |
|  | AC | 98(0.258) | 103(0.275) | 0.587 | 0.914(0.662-1.263) |
|  | CC | 4(0.011) | 7(0.019) | 0.348 | 0.558(0.162-1.921) |
|  | A | 654(0.861) | 631(0.844) | 0.354 | 1.144(0.861-1.521) |
|  | C | 106(0.139) | 117(0.156) | 0.354 | 0.874(0.658-1.162) |
| rs205500 | TT | 270(0.711) | 275(0.733) | 0.484 | 0.893(0.649-1.228) |
|  | TC | 97(0.255) | 95(0.253) | 0.951 | 1.010(0.728-1.402) |
|  | CC | 13(0.034) | 5(0.013) | 0.060 | 2.621(0.925-7.427) |
|  | T | 637(0.838) | 645(0.860) | 0.236 | 0.843(0.636-1.118) |
|  | C | 123(0.162) | 105(0.140) | 0.236 | 1.186(0.894-1.573) |
| rs2525997 | AA | 73(0.194) | 54(0.144) | 0.064 | 1.437(0.977-2.112) |
|  | AC | 164(0.436) | 167(0.444) | 0.826 | 0.968(0.726-1.291) |
|  | CC | 139(0.370) | 155(0.412) | 0.232 | 0.836(0.624-1.121) |
|  | A | 310(0.412) | 275(0.366) | 0.064 | 1.217(0.988-1.497) |
|  | C | 442(0.588) | 477(0.634) | 0.064 | 0.822(0.668-1.012) |
| rs58489644 | AA | 265(0.701) | 260(0.691) | 0.775 | 1.046(0.767-1.427) |
|  | AG | 95(0.251) | 106(0.282) | 0.342 | 0.855(0.619-1.181) |
|  | GG | 18(0.048) | 10(0.027) | 0.127 | 1.830(0.833-4.018) |
|  | A | 625(0.827) | 626(0.832) | 0.767 | 0.960(0.734-1.256) |
|  | G | 131(0.173) | 126(0.168) | 0.767 | 1.041(0.796-1.362) |
| rs138059750 | AA | 377(1.000) | 367(0.992) | 0.121 | - |
|  | AC | 0(0) | 3(0.008) | 0.121 | - |
|  | CC | 0(0) | 0(0) | - | - |
|  | A | 754(1.000) | 737(0.996) | 0.121 | - |
|  | C | 0(0) | 3(0.004) | 0.121 | - |
| rs77107579 | TT | 324(0.871) | 338(0.887) | 0.496 | 0.859(0.554-1.332) |
|  | TC | 46(0.124) | 41(0.108) | 0.491 | 1.170(0.748-1.831) |
|  | CC | 2(0.005) | 2(0.005) | 1.000 | 1.024(0.144-7.310) |
|  | T | 694(0.933) | 717(0.941) | 0.515 | 0.871(0.575-1.321) |
|  | C | 50(0.067) | 45(0.059) | 0.515 | 1.148(0.757-1.740) |

VKH: Vogt-Koyanagi-Harada Disease; OR: odds ratio; 95% CI: 95% confidence interval.

*P* < 2.08×10^-3^ indicates statistical significance.

**Supplementary Table S4. Stratified analysis for rs7779972 with the major clinical features of VKH disease in the combined study.**

| Clinical features | Genotype/  Allele | VKH  with (%) | VKH without (%) | *P* | OR (95%CI) |
| --- | --- | --- | --- | --- | --- |
| Sunset glow fundus | AA | 35(9.9) | 56(14.7) | 0.048 | 0.637(0.406-0.998) |
|  | AG | 166(46.8) | 172(45.0) | 0.637 | 1.072(0.802-1.433) |
|  | GG | 154(43.4) | 154(40.3) | 0.399 | 1.134(0.846-1.521) |
|  | A | 236(33.2) | 284(12.8) | 0.114 | 0.842(0.679-1.043) |
|  | G | 474(66.8) | 480(45.3) | 0.114 | 1.188(0.959-1.472) |
| Headache | AA | 41(11.5) | 50(13.1) | 0.526 | 0.867(0.558-1.347) |
|  | AG | 167(47.0) | 171(44.8) | 0.535 | 1.096(0.820-1.465) |
|  | GG | 147(41.4) | 161(42.1) | 0.839 | 0.970(0.724-1.300) |
|  | A | 249(35.1) | 271(35.5) | 0.872 | 0.983(0.793-1.217) |
|  | G | 461(64.9) | 493(64.5) | 0.872 | 1.018(0.822-1.260) |
| Tinnitus | AA | 39(12.0) | 52(12.6) | 0.799 | 0.944(0.606-1.471) |
|  | AG | 147(45.2) | 191(46.4) | 0.760 | 0.956(0.714-1.279) |
|  | GG | 139(42.8) | 169(41.0) | 0.633 | 1.075(0.800-1.443) |
|  | A | 225(34.6) | 295(35.8) | 0.636 | 0.949(0.765-1.178) |
|  | G | 425(65.4) | 529(64.2) | 0.636 | 1.053(0.849-1.307) |
| Dysacusia | AA | 36(14.0) | 55(11.5) | 0.316 | 1.259(0.802-1.975) |
|  | AG | 110(42.8) | 228(47.5) | 0.222 | 0.827(0.610-1.122) |
|  | GG | 111(43.2) | 197(41.0) | 0.573 | 1.092(0.804-1.484) |
|  | A | 182(35.4) | 338(35.2) | 0.939 | 1.009(0.806-1.262) |
|  | G | 332(64.6) | 622(64.8) | 0.939 | 0.991(0.792-1.240) |
| Alopecia | AA | 30(13.2) | 61(12.0) | 0.633 | 1.121(0.702-1.790) |
|  | AG | 101(44.5) | 237(46.5) | 0.619 | 0.923(0.674-1.264) |
|  | GG | 96(42.3) | 212(41.6) | 0.854 | 1.030(0.750-1.414) |
|  | A | 161(35.5) | 359(35.2) | 0.921 | 1.012(0.803-1.275) |
|  | G | 293(64.5) | 661(64.8) | 0.921 | 0.988(0.784-1.246) |
| Poliosis | AA | 22(10.5) | 69(13.1) | 0.344 | 0.783(0.470-1.302) |
|  | AG | 101(48.3) | 237(44.9) | 0.398 | 1.148(0.833-1.583) |
|  | GG | 86(41.1) | 222(42.0) | 0.824 | 0.964(0.696-1.334) |
|  | A | 145(34.7) | 375(35.5) | 0.766 | 0.965(0.761-1.223) |
|  | G | 273(65.3) | 681(64.5) | 0.766 | 1.037(0.818-1.315) |
| Vitiligo | AA | 8(9.2) | 83(12.8) | 0.341 | 0.692(0.323-1.483) |
|  | AG | 40(46.0) | 298(45.8) | 0.982 | 1.005(0.642-1.575) |
|  | GG | 39(44.8) | 269(41.4) | 0.541 | 1.151(0.733-1.806) |
|  | A | 56(32.2) | 464(35.7) | 0.363 | 0.855(0.610-1.199) |
|  | G | 118(67.8) | 836(64.3) | 0.363 | 1.170(0.834-1.639) |

VKH: Vogt-Koyanagi-Harada Disease. OR: odds ratio; 95% CI: 95% confidence interval.

*P* < 2.08×10^-3^ indicates statistically significant.

**Supplementary Table S5. Minor allele frequencies of rs7779972 in VKH patients and different populations’ controls.**

| Populations | Controls  (n) | MAF  (cases/ controls) | HWE | P | OR (95% CI) |
| --- | --- | --- | --- | --- | --- |
| EAS | 504 | 0.353/0.340 | 0.03 | 0.52 | 1.057(0.893-1.251) |
| CDX | 93 | 0.353/0.382 | 0.13 | 0.44 | 0.883(0.645-1.209) |
| CHB | 103 | 0.353/0.325 | 0.96 | 0.44 | 1.131(0.829-1.543) |
| CHS | 105 | 0.353/0.319 | 0.02 | 0.34 | 1.163(0.854-1.585) |
| JPT | 104 | 0.353/0.337 | 0.73 | 0.65 | 1.075(0.791-1.460) |
| KHV | 99 | 0.353/0.343 | 0.30 | 0.80 | 1.042(0.763-1.424) |

VKH: Vogt-Koyanagi-Harada Disease; HWE: Hardy–Weinberg equilibrium; OR: odds ratio; 95% CI: 95% confidence interval. MAF: minor allele frequency.

EAS: East Asian; CDX: Chinese Dai in Xishuangbanna, China; CHB: Han Chinese in Beijing, China; CHS: Southern Han Chinese, China; JPT: Japanese in Tokyo, Japan; KHV: Kinh in Ho Chi Minh City, Vietnam.

*P* < 0.05 indicates statistically significant.
